# Supplementary material for: DNA topoisomerase 3 is required for efficient germ cell quality control
Source: J Cell Biol. 2021 Apr 2;220(6):e202012057. doi: 10.1083/jcb.202012057 (PMC8025215; doi:10.1083/jcb.202012057)
Supplement: Table S1 — lists reagents and resources. [file JCB_202012057_TableS1.docx]

**Reagents and Resources Table**

| REAGENT or RESOURCE | SOURCE | IDENTIFIER |
| --- | --- | --- |
| **Antibodies** | | |
| Anti-RAD-51 | Zetka | N/A |
| Anti-pCDK-1 | Calbiochem | Cat. # 219440 |
| Anti-CEP-1 | A. Gartner | N/A |
| Anti-SIR2.1 | A. Gartner | Greiss et al., 2008 |
| Anti-PGL-1 | A. Hyman | N/A |
| Anti-digoxigenin-conjugated with rhodamine | Roche | Cat. #11207750910 |
| Anti-biotin | Abcam | Cat. # ab6650 |
| Anti-HA | Sigma-Aldrich | Cat. # H6908 |
| **Chemicals, Peptides and Recombinant Proteins** | | |
| SYTO-12 | Thermo Fischer Scientific | Cat. # S7574 |
| Vectashield Mounting Medium | Vector Laboratories | Cat. #H-1000 |
| **Critical Commercial Assays** | | |
| Digoxigenin-nick translation Kit | Sigma-Aldrich | Cat. #11745816910 |
| Biotin-Nick Translation Kit | Sigma-Aldrich | Cat. #11745824910 |
| Bioline SensiFAST SYBR No-ROX Kit | Labconsulting | Cat. #Bio-98005 |
| PeqGOLD TriFast | Peqlab, VWR | Cat. #30-2010 |
| MEGAscript T3 Transcription Kit | Ambion, Invitrogen | Cat. #AM1338 |
| SuperScript III Frist-strand Synthesis system | Invitrogen | Cat. #18080051 |
| 3-Indoleacetic acid | Sigma-Aldrich | Cat. #I3750-25G-A |
| Zero blunt TOPO PCR Cloning Kit | Invitrogen | Cat. #45-0245 |
| Click-iT™ EdU Cell Proliferation Kit for Imaging, Alexa Fluor™ 555 dye | Invitrogen | Cat. # C10338 |
| **Experimental Models: Organisms/Strains** | | |
| *C. elegans*: N2 Bristol | CGC | https://cgc.umn.edu/strain/search |
| *C. elegans*: *top-3 jf101[Y56A3A.27::unc-119(+)]/hT2 (I;III)* | This paper | UV149 |
| *C. elegans*: *spo-11(ok79)/nT1* *(IV;V)* | (Dernburg et al., 1998) | AV106 |
| *C. elegans*: *spo-11(ok79)/nT1 IV; +/nT1 V.top-3 jf101[Y56A3A.27::unc-119(+)] oxTi539[eft-3p::TdTomato::H2B::unc-54 3ÚTR + Cbr-unc-119(+) ] III/ eft-3p::GFP III* | This paper | UV150 |
| *C. elegans*: *rfs-1(ok1372) III* | CGC | RB1279 |
| *C. elegans*: *rfs-1 (ok1372) top-3 jf101[Y56A3A.27::unc-119(+)]/hT2 (I;III)* | This paper | UV151 |
| *C. elegans*: *bcIs39[P(lim-7)ced-1::GFP + lin-15(+)]* | (Zhou et al., 2001) | MD701 |
| *C. elegans*: *bcIs39[P(lim-7)ced-1::GFP + lin-15(+)];top-3 jf101[Y56A3A.27::unc-119(+)]/hT2 (I;III)* | This paper | UV152 |
| *C. elegans*: *prom-1(ok1140) unc-55 LGI /hT2[bli-4(e937) let-?(q782) qIs48] (I;III), bcIs39[P(lim-7)ced-1::GFP + lin-15(+)]* | This paper | UV202 |
| *C. elegans: prom-1(ok1140) I* | (Jantsch et al., 2007) | RB1183 |
| *C. elegans*: *rad-51::degron (jf121)IV unc-119(ed3) III; ieSi38 [Psun-1 TIR-1::mRuby::sun-1 3ʹUTR, cb-unc-119(+)] IV* | This paper | UV154 |
| *C. elegans*: *top-3 jf101[Y56A3A.27:::unc-119(+)]/hT2 (I;III); rad-51::degron (jf121)IV unc-119(ed3) III; ieSi38 [Psun-1 TIR-1::mRuby::sun-1 3ʹUTR, cb-unc-119(+)] IV* | This paper | UV155 |
| *C. elegans*: *cku-80(ok861)* | (Checchi et al., 2014) | RB964 |
| *C. elegans*: *top-3 jf101[Y56A3A.27::unc-119(+)]/hT2 (I;III); cku-80 (ok861)III(-/-)* | This paper | UV156 |
| *C. elegans*: *cep-1(gk138) I* | CGC | TJ1 |
| *C. elegans*: *cep-1(gk138) I./ht2; top-3(jf101[Y56A3A.27::unc-119(+)])/ht2 (I;III)* | This paper | UV157 |
| *C. elegans*: *cep-1(gk138)/ht2(I); top-3(jf101) [Y56A3A.27::unc-119(+)])/ht2(III) cku-80(OK861) III (-/-)* | This paper | UV158 |
| *C. elegans*: *gfp::cku-80(jf150)* | This paper | UV159 |
| *C. elegans*: *top-3(jf101)[Y56A3A.27::unc-119(+)]/ht2(I;III) gfp::cku-80(jf150)III (-/-)* | This paper | UV160 |
| *C. elegans: gfp::cku-80(jf150) unc-32(e189) com-1(t1626)/ht2 gfp::cku-80(jf150) III* | This paper | UV203 |
| *C. elegans*: *brc-1(tm1145) III* | CGC | DW102 |
| *C. elegans*: *top-3 jf101[Y56A3A.27::unc-119(+)]/hT2 (I;III)brc-1(tm1145) III(-/-)* | This paper | UV162 |
| *C. elegans*: *polq-1 (tm2026)* | National BioResource Project | W03A3.2 |
| *C. elegans*: *polq-1 (tm2026) top-3 jf101[Y56A3A.27::unc-119(+)]/hT2 (I;III)* | This paper | UV163 |
| *C. elegans*: *xpf-1(tm2842) II; top-3 jf101[Y56A3A.27::unc-119(+)]/hT2 (I;III)* | This paper | UV165 |
| *C. elegans*: *xpf-1(tm2842) II* | (Lans et al., 2013) | TG1660 |
| *C. elegans*: *mus-81 (tm1937) I* | (Kruisselbrink et al., 2008) | TG1760 |
| *C. elegans*: *mus-81(tm1937) I /hT2,(I;III);top-3 (jf101)[Y56A3A.27::unc-119(+)]/ hT2,(I;III)* | This paper | UV166 |
| *C. elegans*: *zhp-3(jf61) I; top-3( jf101)[Y56A3A.27::unc-119(+)]/hT2 (I;III)* | This paper | UV167 |
| *C. elegans*: *zhp-3(jf61::unc-119+)/+ I; unc-119(ed3) III* | (Jantsch et al., 2004) | UV1 |
| *C. elegans*: *zhp-3(jf61) I; brc-1(tm1145)III -/- top-3( jf101)[Y56A3A.27::unc-119(+)]/hT2 (I;III)* | This paper | UV168 |
| *C. elegans*: *opIs263 [rpa-1p::rpa-1::YFP + unc-119(+)] III* | (Stergiou et al., 2011) | WS4581 |
| *C. elegans: opIs263 [rpa-1p::rpa-1::YFP + unc-119(+)] top-3 jf101[Y56A3A.27::unc-119(+)]/ht2(III;I)* | This paper | UV173 |
| *C. elegans: rmh-1(jf54) I* | (Jagut et al., 2016) | UV173 |
| *C. elegans: him-6 (ok412) IV* | (Wicky et al., 2004) | VC193 |
| *C. elegans: rmh-2 (jf94[T07C12.12 ::unc-119+]) V* | (Jagut et al., 2016) | UV174 |
| *C. elegans:* *cku-70 (tm1524) top-3 jf101[Y56A3A.27:::unc-119(+)] III /hT2 (I;III)* | This paper | UV200 |
| *C. elegans: cku-70 (tm1524) III* | CGC | FX1524 |
| *C. elegans:* *top-3 jf101[Y56A3A.27:::unc-119(+)]lig-4(ok716) III/hT2 (I;III)* | This paper | UV201 |
| *C. elegans: lig-4(ok716) III* | (Kruisselbrink et al., 2008) | RB873 |
| *C. elegans: glp-1(bn18) III* | (Kodoyianni et al., 1992) | DG2389 |
| *C. elegans; top-3 jf101[Y56A3A.27::unc-119(+)] cku-70 (tm1524) polq-1 (tm2026)/hT2 (I;III)* | This paper | UV206 |
| *C. elegans; cku-70 (tm1524) polq-1 (tm2026) III* | This paper | UV205 |
| *C. elegans; top-3::ha::degron(jf122) III unc-119(ed3) III; ieSi38 [Psun-1 TIR-1::mRuby::sun-1 3ʹUTR, cb-unc-119(+)] IV* | This paper | UV207 |
| *C. elegans; top-3::ha::degron(jf122) III unc-119(ed3) III; ieSi38 [Psun-1 TIR-1::mRuby::sun-1 3ʹUTR, cb-unc-119(+)] IV him-6(ok421) /nt1 IV* | This paper | UV233 |
| *C. elegans; top-3::ha::degron(jf122) III cku-80(ok861) III unc-119(ed3) III; ieSi38 [Psun-1 TIR-1::mRuby::sun-1 3ʹUTR, cb-unc-119(+)] IV* | This paper | UV234 |
| **Recombinant DNA** | | |
| Destination vector pDESTttTi5605[R4-R3] for MOS insertion on Chromosome II (was a gift from Erik Jorgensen) | (Frokjaer-Jensen et al., 2008) | pCFJ150  Addgene plasmid # 19329 |
| Co-injection marker *Pmyo-2::mCherry::unc-54utr* (was a gift from Erik Jorgensen) | (Frokjaer-Jensen et al., 2008) | pCFJ90  Addgene plasmid # 19327 |
| Co-injection marker pGH8 - *pRAB-3::mCherry::unc-54utr* (was a gift from Erik Jorgensen) | (Frokjaer-Jensen et al., 2008) | pGH8  Addgene plasmid # 19359 |
| *Peft-3::cas9-SV40_NLS::tbb-2 3ʹUTR* (was a gift from John Calarco) | (Friedland et al., 2013) | Addgene plasmid # 46168 |
| *PU6::klp-12*_sgRNA (was a gift from John Calarco) | (Friedland et al., 2013) | Addgene plasmid # 46170 |
| **Software and Algorithms** | | |
| ImageJ version 2.0.0 | NIH | https://imagej.nih.gov/ij/ |
| SoftWoRx Suite | Applied Precision | N/A |
| GraphPad Prism 6 | GraphPad | N/A |
| Adobe Photoshop CC2018 | Adobe | N/A |
| Adobe Illustrator CC2018 | Adobe | N/A |

Dernburg, A.F., K. McDonald, G. Moulder, R. Barstead, M. Dresser, and A.M. Villeneuve. 1998. Meiotic recombination in C. elegans initiates by a conserved mechanism and is dispensable for homologous chromosome synapsis. *Cell*. 94:387-398.

Frokjaer-Jensen, C., M.W. Davis, C.E. Hopkins, B.J. Newman, J.M. Thummel, S.P. Olesen, M. Grunnet, and E.M. Jorgensen. 2008. Single-copy insertion of transgenes in Caenorhabditis elegans. *Nat Genet*. 40:1375-1383.

Kodoyianni, V., E.M. Maine, and J. Kimble. 1992. Molecular basis of loss-of-function mutations in the glp-1 gene of Caenorhabditis elegans. *Mol Biol Cell*. 3:1199-1213.

Kruisselbrink, E., V. Guryev, K. Brouwer, D.B. Pontier, E. Cuppen, and M. Tijsterman. 2008. Mutagenic capacity of endogenous G4 DNA underlies genome instability in FANCJ-defective C. elegans. *Curr Biol*. 18:900-905.

Zhou, Z., E. Hartwieg, and H.R. Horvitz. 2001. CED-1 is a transmembrane receptor that mediates cell corpse engulfment in C. elegans. *Cell*. 104:43-56.
